# Barriers and Motivators to Opioid Treatment Among Suburban Women Who Are Pregnant and Mothers in Caregiver Roles

**DOI:** 10.3389/fpsyg.2021.688429

**Published:** 2021-07-01

**Authors:** Miriam Boeri, Aukje K. Lamonica, Jeffrey M. Turner, Amanda Parker, Grace Murphy, Carly Boccone

**Affiliations:** ^1^Department of Sociology, Bentley University, Waltham, MA, United States; ^2^Department of Public Health, Southern Connecticut State University, New Haven, CT, United States

**Keywords:** opioid treatment, pregnant women, mothers, motivators, facilitators, barriers

## Abstract

Women of childbearing age who misuse opioids are a particularly vulnerable population, and their barriers to treatment are unique because of their caregiver roles. Research on treatment for opioid use generally draws from urban and rural areas. This study fills a gap in research that focuses on barriers and motivators to opioid treatment in suburban areas. The aim of this study was to give voice to suburban pregnant women and mothers caring for children while using opioids. Ethnographic methods were used for recruitment, and 58 in-depth interviews were analyzed using a modified grounded theory approach. Barriers to medication-assisted treatment (MAT) included stigma, staff attitudes, and perceptions the women had about MAT treatment. Barriers associated with all types of treatment included structural factors and access difficulties. Relationships with partners, friends, family, and providers could be barriers as well as motivators, depending on the social context of the women’s situation. Our findings suggest increasing treatment-seeking motivators for mothers and pregnant women by identifying lack of resources, more empathetic consideration of social environments, and implementing structural changes to overcome barriers. Findings provide a contemporary understanding of how suburban landscapes affect mothers’ treatment-seeking for opioid dependence and suggest the need for more focus on emotional and structural resources rather than strict surveillance of women with opioid dependence who are pregnant or caring for children.

## Introduction

Over the last two decades, drug overdose deaths have more than quadrupled in number (Scholl et al., 2019). Between 2010 and 2017, opioid-related overdose deaths increased five-fold (Hedegaard et al., 2019). After a slight decrease in overdose death rates from 2017 to 2018, the introduction of synthetic fentanyl into the United States drug market resulted in a sharp rise of overdose deaths during 2019 (Lambdin et al., 2019). Recent reports from the Centers for Disease Control and Prevention (CDC) suggest COVID-19 is accelerating overdose death trends (Centers for Disease Control and Prevention (CDC), 2020). Failures in the management of the opioid crisis were compounded after COVID-19 disrupted services, resulting in more barriers to opioid treatment (del Pozo and Beletsky, 2020; Haley and Saitz, 2020).

The opioid problem in the United States began with healthcare providers overprescribing prescription opioids (Okie, 2010; Kolodny et al., 2015; Humphreys, 2017; Ciccarone, 2019). Data show that women fill more prescriptions than men, and women are more likely than men to be given a prescription by their provider (Centers for Disease Control and Prevention (CDC), 2018; Hirschtritt et al., 2018; Marsh et al., 2018; Becker and Mazure, 2019). The CDC reports that use of prescription (oxycodone, hydrocodone) and illegal (fentanyl, heroin) opioids has risen steadily among women of reproductive age (15–44) throughout the past decade, and deaths from opioid overdose increased nearly 500% among women, more than double the rate among men (Centers for Disease Control and Prevention (CDC), 2018; Mazure and Fiellin, 2018). We know that medical opioid use serves as a stepping-stone to the use of heroin and illegal opioid analogs, creating greater risk for overdose events (Vuong et al., 2010; Whiteman et al., 2014; Kolodny et al., 2015). During the commercial lockdown and social isolation policies implemented to address the coronavirus pandemic, opioid overdose incidents increased again, particularly among minority and vulnerable populations (Ochalek et al., 2020; Slavova et al., 2020; Sun et al., 2020).

Women of childbearing age who misuse opioids are a particularly vulnerable population as they juggle their own substance dependence, pregnancy, and motherhood. To address the rising rates of overdose morbidity and mortality, there has been a shift toward medication-assisted treatment (MAT) for opioid dependence (Scholl et al., 2019; Abraham et al., 2020; Adams and Volkow, 2020). MAT has become the gold standard for opioid dependence in pregnant women (Timmermans and Berg, 2010; Klaman et al., 2017; Reddy et al., 2017; Verduin, 2017). MAT used in the United States include methadone, an opioid agonist, burprenorphine, a partial opioid agonist, and naloxone and naltrexone, which are opioid antagonists. Although pregnant women are recommended methadone, providers also recommend MAT such as Suboxone, which includes both agonistic and antagonistic properties (Meyer et al., 2015). MAT like all drug treatment for mothers is concomitant with assertive child protective interventions (Cochran et al., 2018; Lacaze-Masmonteil and O’Flaherty, 2018; Murphy et al., 2018), and fear of intensified scrutiny from healthcare providers leads women to hide their use or relapse when resources needed to cope with life stressors are not provided (Woodall and Boeri, 2013; Goodman et al., 2019; Whittaker et al., 2019; Lamonica et al., 2021).

The barriers to treatment for mothers are unique because of their caregiver roles, and they often fear government intrusion will result in loss of their custodial rights as parents (Paltrow et al., 2004; Howard, 2016; Dondorp and de Wert, 2017). Increased surveillance and stigmatization by medical staff and law enforcement present additional barriers to seeking necessary treatment (McMahon et al., 2002; Paltrow and Flavin, 2013; Olsen, 2015; Angelotta et al., 2016; Frazer et al., 2019; Honein et al., 2019).

Stigmatization is the process of labeling and stereotyping that often leads to social rejection, exclusion, and isolation, as well as internalizing discrediting attitudes perceived in others (Goffman, 1959; Chaudoir et al., 2013). Fear of stigmatization discourages women from seeking help and engaging in treatment (VanDeMark, 2007; Radcliffe, 2011; Stone, 2015). Losing custody of their children due to opioid use adds to feelings of shame and guilt, as social stigmatization increases (Howard, 2015; Knight, 2015; Lee and Boeri, 2017; Nichols et al., 2021). Parental drug abuse is the reason associated with one-third of child removal cases in the United States in 2019 (U.S. Department of Health and Human Services, 2020), creating an incentive for mothers to keep their drug use hidden (Angelotta et al., 2016).

Previous studies identified common barriers to drug treatment that include costs, access, waiting lists, institutionalized stigma, transportation, lack of social support, and isolation (Pollini et al., 2006; Redko et al., 2006; Wisdom et al., 2011; Harris and McElrath, 2012; Hammarlund et al., 2018; Kahn et al., 2019; Acevedo et al., 2020). Barriers specific to mothers also include lack of childcare services and difficulties in relational situations (Marsh et al., 2000; Frazer et al., 2019). Findings on barriers to treatment far outweigh findings on facilitators to treatment (Wisdom et al., 2011), and research on facilitators tends to focus on individual traits, such as mental health, motivation, and treatment readiness (Rapp et al., 2007; Hiller et al., 2009). Treatment readiness research on women who are mothers or pregnant is scarce (Frazer et al., 2019), and research on the impact of treatment for pregnant women with opioid dependence is evolving (McCarthy et al., 2017; Rizk et al., 2019). Research on opioid use generally focuses on urban areas or rural communities, including research on treatment for women with children (Marsh et al., 2000; Young et al., 2010; Jonas et al., 2012; Wang et al., 2013; Frazer et al., 2019; Ochalek et al., 2020). In this study, we fill a gap in research that focuses on both barriers and motivators to opioid treatment among pregnant women and custodial mothers who live in suburban areas.

In the past, suburban communities were not viewed as high-risk areas for drug research or drug treatment funding. Reports on increased opioid use and opioid-related overdose mortality rates in the suburbs (Cicero et al., 2014; Kuehn, 2014) only recently drew greater awareness of the suburbs as a risk environment for opioid use (Zoorob and Salemi, 2017; Boeri and Lamonica, 2020). Suburban towns have fewer treatment programs for women and other needed health and social services compared to cities, and residential treatment in the suburbs for women with children in their care is virtually non-existent (Allard and Roth, 2010; Lamonica et al., 2021). Reports of increased opioid dependence among women with children and pregnant women reveal an urgent need for treatment that addresses the social and familial situation of suburban mothers (Marsh et al., 2000; Maeda et al., 2014; Patrick et al., 2015; Angelotta et al., 2016; Klaman et al., 2017; Reddy et al., 2017; Boeri and Lamonica, 2020; Lamonica et al., 2021).

In this paper, we provide a contemporary understanding of how suburban landscapes affect mothers’ treatment-seeking for opioid dependence. As a qualitative study, we provide verbatim perspectives from women who were using opioids while pregnant or rearing children. Our aim is to understand the factors that motivate or discourage treatment-seeking behaviors among these women in order to inform opioid treatment and associated healthcare and social services for pregnant women and mothers of young children living in the suburbs.

## Materials and Methods

The data analyzed for this paper were drawn from the Suburban Opioid Study (SOS). The goal of the study was to fill a gap in our understanding of opioid use patterns in suburban communities where overdose mortality rates were increasing. Qualitative and quantitative data were collected using audio-recorded in-depth interviews and life history surveys. The Institutional Review Board (IRB) from the investigators’ academic institutions approved the study, and a “Certificate of Confidentiality” was obtained from a federal agency to protect study data and researchers from *sub poena*. Data were collected between June 2017 and July 2019. The study sites were the suburban towns around Atlanta, Georgia; Boston, Massachusetts; and New Haven, Connecticut.

### Recruitment and Participants

Ethnographic fieldwork was used to provide direct access to people who used opioids. Fieldwork consisted of spending time in areas where drug use or drug selling were observed, developing rapport with community members, and leaving study fliers with our phone number in strategic places (e.g., laundromats, bus stations, fast food restaurants, harm reduction centers) (Page and Singer, 2010). The fieldwork was occasionally aided by community consultants, who are people in the community who have knowledge of use patterns and settings of opioid use. Targeted and snowball sampling methods were used to increase diversity of race and gender (Biernacki and Waldorf, 1981; Watters and Biernacki, 1989). Eligibility criteria included (1) having misused/abused opioids at least once in the last month, (2) resided in a suburban location, and (3) were 18 years of age or older. Of the 173 interviews collected in the larger study, females represented 44.5% of the sample.

This paper is based on interviews conducted with 58 women drawn from the SOS sample who were using opioids during a time when they were pregnant or taking care of children in their custody. Table 1 provides the demographic and social context of the women who were part of the analysis for this paper. The women ranged in age from 25 to 63 with a mean of 42.8 years. Among the sample of mothers, 63.8% were White, 20.8% identified as African American/Black, and 15.5% as Latina. Forty-four women had been involved with the criminal justice system and 45 had been homeless at some time in their lives. Almost 90% said they had been in treatment, often multiple times. The types of treatment that women experienced included MAT (82.8%), 12-step meetings (36.2%) and residential treatment (34.5%). Women discussed outpatient treatment in reference to MAT and 12-step meetings; therefore, outpatient is not distinguished in the table. Six of the women had not been in structured treatment but said they experienced barriers to entering treatment during pregnancy and child-rearing.

**TABLE 1:** Participant demographic and social information (*N* = 58).

**Characteristic**	**M (range) or% (*n*)**
*Age* Mean (range)	42.8 (25–63)
***Race/Ethnicity***	
White	63.8 (37)
African-American/Black	20.7 (12)
Latina/White	8.6 (5)
Latina/Black	1.7 (1)
Latina/Other	5.2 (3)
*Ever CJ involved*	75.8 (44)
*Ever Homeless*	77.6 (45)
*Ever in Treatment*	89.7 (52)
*Types of Treatment*	
MAT	82.8 (48)
12-Step	36.2 (21)
Residential	34.5 (20)
None	10.3 (6)

### Data Collection

Interviews were conducted in participants’ homes, private offices, library rooms, fieldworkers’ cars, parks, and other quiet places in private or public spaces. Participants were provided a consent form to read before the interview that explained study procedures, risks, and benefits. Participants gave oral consent that was audio-recorded so signatures were not required on the consent form. At no point were the participants asked to provide identifying information, such as names, addresses, or phone numbers. The audio-recorded interviews were transcribed with instructions to delete any identifying material that may have been said inadvertently. All data were further anonymized to ensure no identifying information remained.

Participants received $40 for their time at the end of the interview. Interviews lasted between two to four hours. Long interviews are typical in qualitative research conducted in environments where participants feel safe and comfortable. We used a participant-focused interview style in which a semi-structured interview guide provided questions, but we allowed participants to take the interview in different directions. Interviewees were asked to refer potential participants to call the study phone number for a small referral fee.

### Data Analysis

The data analyzed for this paper focused on the sections of the qualitative interview in which the women talked about their feelings toward opioid treatment, experiences with different treatment modalities, reasons for seeking or participating in treatment, and perspectives on maintaining a treatment regime while pregnant or caring for children. While grounded theory methods have developed in different directions by the creators of this method (Glaser and Strauss, 1967; Strauss and Corbin, 1998), here we use a modified grounded theory approach, which allows for only parts of the transcripts to be coded and not a line-by-line analysis of the entire interview (Charmaz, 2014). Grounded theory is responsive to subjective meanings revealed by participants during the interview and meanings that emerge during analysis. Other parts of the women’s interviews were used to provide more clarity or context to treatment seeking.

As is common in grounded theory methods, data analysis and data collection are conducted simultaneously (Strauss and Corbin, 1998). Coding began before all interviews were collected. The process for identifying themes was dynamic, as new codes emerged from the data. Each transcript was read and coded by at least two authors of this paper and many were coded by three. Final coding occurred after all data were collected. Coding began by reading the transcripts to identify key themes and conceptual categories underlying the subjective meanings revealed in the women’s narratives. All transcripts were entered into NVivo, a software program for organizing qualitative data to make them more manageable and to enhance the reliability of the results.

Trustworthiness of the coding was achieved by frequent meetings among the authors in which emerging findings were dissected and reviewed for legitimacy using “mixed-methods triangulation” as well as “theoretical triangulation” (Renz et al., 2018, p. 827). Triangulation refers to using more than one method for data collection, or more than one theory when analyzing and interpreting qualitative data. Our mixed-methods analysis combined in-depth interviews and brief surveys to increase confidence in the data and trustworthiness of the interpretation (Plano Clark, 2010; Laenen, 2011). The qualitative data sources informing the analysis included transcripts of audio-recorded in-depth interviews, field notes, memos, and quantitative data collected with surveys. The theoretical frameworks that guided the analysis included social stigma and life course theories (Goffman, 1959; Elder, 1999; Harris and McElrath, 2012; Chaudoir et al., 2013; Howard, 2015; Nichols et al., 2021). A life course perspective helps to unravel the effect of structural constraints from situational contexts that change over time by focusing attention on transitions and turning points (Elder, 1999; Hser et al., 2007; Whalen and Boeri, 2014). Life course analysis provides insights on the interaction between social bonding mechanisms, such as relationships, and social control (Laub and Sampson, 2003), as well as the interactional processes between emotions and social control (Collins, 2004). Consistent with grounded theory analysis, a triangulation of these theories was used to identify themes and patterns in the data to develop knowledge of new phenomena that move beyond one theoretical framework (Glaser and Strauss, 1967; Charmaz, 2014).

All codes and concepts were discussed among the authors to compare definitions, assess illustrative quotes, and ensure consistency of meaning. Categories were re-examined, defined, fragmented, or integrated into two guiding concepts: *barriers* and *motivators* to engaging in treatment. Barriers to treatment were greater in terms of variety and number of obstacles, and in terms of the depth of difficulties that are unique to women who use opioids while they are pregnant or have children in their custody. Motivators could be barriers depending on the situation or circumstances of the women.

The results of this analysis are supported by quotes from the women that are verbatim except when an ellipsis is inserted in brackets [.] to indicate words are deleted that do not change the meaning of the quote. Words are inserted in brackets to protect the anonymity of participants. All names are pseudonyms. Child Protective Services () is called by different names in the three states. To protect anonymity, we use CPS regardless of the state where the mother lived.

## Results

We uncovered several barriers to seeking opioid treatment in our qualitative interviews. The life history data used in the analysis relate to when women were pregnant or caring for small children while they were opioid dependent. This means that some of the incidents discussed were before contemporary recommendations to provide MAT to pregnant women; yet, many of the barriers discussed by our participants focused on contemporary access to MAT. These include the social and structural stigma associated with using MAT, clinic staff’s attitudes toward patients, perceptions and pharmacological effects of MAT, and the procedures and operating times of the treatment facilities. Other barriers that were not specific to MAT treatment included treatment facility related barriers such as access for women, costs, and location. We uncovered several factors in the women’s lives that acted as potential barriers or motivators to seeking treatment. Relationships with romantic partners and family or friends could either be helpful or harmful to recovery. Similarly, pregnancy was sometimes a motivator and other times a barrier to treatment. Lastly, the complicated relationships our respondents had with CPS either prompted treatment or led mothers to hide their use and avoid treatment.

### Medication-Assisted Treatment Barriers

Nearly 83% of our participants had experience with using MAT at some point in their lives, which has become more accessible in the past decade; however, this type of treatment also presented challenges. Among the barriers discussed by our participants are stigma of using MAT, the clinic staff’s attitudes, perceptions and pharmacological effects of MAT, and the procedures and operating times of the treatment facilities.

#### MAT Stigma

The majority of the women in our study participated in MAT at some point in the past and relapsed. A common barrier to returning to MAT was the stigma attached to these programs. Some participants experienced stigma by healthcare providers who were not involved in their treatment for their opioid use. Annie, a White mother of four in her 30s, was once motivated to seek treatment to retain her mother role but was now discouraged from seeking MAT because of how stigmatized she perceived this treatment to be. Annie shared her thoughts:

Me and my husband were talkin’…the stigma about the methadone. I just—he’s gonna go and he’s gonna get on the methadone. He’s gonna, but I don’t want to so I don’t know what to do; […]. Because I don’t wanna go to a program every day and I don’t wanna take her there every day. I don’t want a stigma on me. I just want it to be done. I just want it to be over.

Annie’s fear of stigmatization was based on her prior experience in the healthcare setting when she was using methadone. She explained:

I wasn’t treated very nice by certain doctors. And like when I had my baby, (Name), I was on methadone. I felt like the hospital treated me bad. When she was born, I wasn’t on anything and I was treated so much better.

She was adamant about not using methadone to help her treat her opioid use. Krystal, a Black mother in her 50s, concurred with Annie’s hospital experience and added: “It wasn’t the greatest because I’m on methadone so you’re viewed as a drug addict.” Being seen as someone using drugs as opposed to someone using medication was a common complaint among those who had experienced stigmatization.

Stigmatization of MAT use was not always associated with medical providers and staff members of healthcare settings. Some of our participants stigmatized those who used MAT. Here, our example shows that not all MAT were created equal in the minds of our mothers. Particularly methadone was viewed negatively by Vanessa, a White woman in her 30s who considered herself a mother to her spouse’s children. At the time of the interview, she was interested in using Suboxone, the brand name of a MAT composed of buprenorphine (an opioid agonist) and naloxone (an opioid antagonist), to stop her heroin use. She thought “people that are methadone users are finding a cheap way to get high” and casted doubt on their treatment commitment. She insisted that she would never go on methadone.

#### MAT Clinic – Staff Attitudes

Similar to the barriers stigma created for our participants, the behaviors and attitudes of some MAT clinic staff members were discussed as discouraging and identified as barriers to treatment. Several mothers described staff attitudes that negatively influenced their treatment seeking behaviors. One went so far as to just call her doctor at the MAT clinic “an asshole” because of this provider’s demeanor toward her. Likewise, Tess, a White mother of two in her 40s, criticized the staffs’ uncaring attitude:

I just wish that the counselors actually gave a shit. […] If I would miss three days, my counselor would call me and be like, is everything okay? What’s going on? But when I stopped going altogether, I never heard from her. She never once called to say, you haven’t been here in a month; what, what the hell? Are you okay? Are you dead? Nothing.

In Tess’s situation, the behavior of the clinic staff played a role in her not returning to treatment. She wanted the treatment staff to show compassion and care, and when this was not provided, she did not return to treatment. To Tess, treatment was more than a mere dose of methadone, she sought a positive relationship with the provider. She was hoping that the clinic staff would reach out to her to see how she was doing and was deeply disappointed by the lack of follow-up.

Other women took initiative and asked for assistance when they knew they were going to relapse. Mallory, a White mother in her 30s and pregnant at the time of the interview, recently experienced a setback after being sober for four years. She described an episode where she thought she was about to relapse, and she reached out to the methadone clinic for help:

They weren’t helping me. Because I wanted to relapse. I mean they give you. You have to go to a group. They group – you’re not gonna talk in front of 20 people in a group, you’re just not. And I asked my counselor for help; didn’t get it.

Mallory sought someone to talk to who would help her navigate this experience of wanting to relapse. She did not find the group setting that the clinic offered suitable to her needs. In the end, her cravings for heroin became too strong.

Rebecca, a Latina mother of three in her 40s, also struggled with the staff’s attitude at her MAT clinic. She insisted that “they treat you different. It’s always about if you don’t do this, do that, this is what’s gonna happen.” More than anything, Rebecca wished that she had someone to talk to about her opioid use and problems with cessation. At this point, she stated that “I don’t wanna sit down and talk to nobody and tell them this and have groups. I don’t believe in anybody, I don’t trust anybody.” Rebecca desired a more caring clinic environment to support her through the treatment experience, a feeling expressed by other participants.

#### MAT Perception

Some of our participants were hesitant to believe that MAT would be beneficial to them based on their own perceptions and observations. These perceptions were often shaped by hearing others share their negative MAT experiences. Despite being able to afford them, some mothers would not initiate use of MAT. Tiffany, a Black mother of two daughters in her 40s was hiding her opioid use from her physician and husband, fearing that disclosing her use, even with the intention of getting sober, could result in CPS intervention and a divorce. When asked about enrolling in a MAT program, Tiffany described negative perceptions of this kind of treatment: “I don’t wanna do that either because a lot of people tell me that that’s addictive. So, no.” Tiffany feared exchanging one drug with another, and her goal was to wean herself off the opioids. Thus far, that had not been successful.

Tiffany was not alone with thoughts that hindered the utilization of MAT. Vanessa’s perceptions of MAT derived from observing painful methadone withdrawals in other women, and she believed that the opioid medication “does more harm than good.” These observations were complimented by her belief that methadone was just another drug: “I get it’s cheaper. I get that, but the whole point of methadone is to get you off of drugs, when really all it’s doing is getting you off of one and putting you on another.” These observations ultimately led her to say that she would “never go to a methadone clinic.”

#### Pharmacological Effects of MAT

For each MAT modality, there is a wide variety of pharmacological side effects reported in the research literature, and these side effects can range from mild to more severe. Some women in our study experienced adverse pharmacological effects of using MAT or witnessed those effects in others. These experiences affected their willingness to use or continue this method of treatment. Katie, a White woman in her 30s who was motivated to move from a rural area to the suburbs to access MAT, could not continue using Suboxone because it was no longer effective “as a crutch to get through the withdrawal process.” When asked about using methadone, Katie recalled her experience:

But I hated it. I mean it just makes you like so (emphasis on “so”) lethargic and tired. All I wanted to do was sleep. I could not be productive. I could not work a job. I mean I just wanted to lay around and sleep. And I mean I was on a pretty low dose too. I think I was only on like 30 or 40 milligrams a day and I still could not pick my head up.

Katie was now deterred from using MAT because of her experience with the pharmacological side effects of the drug.

Jennifer, a Latina woman in her 40s agreed with Katie. She stopped attending the MAT program and refused to initiate this type of treatment in the future because of its pharmacological effect. She described how methadone made her feel unable to operate a vehicle safely:

I was on the methadone clinic. It was 85 milligrams. I just hopped on it for a minute and got off because I, I just get scared. People are like, what? I don’t know, what if I get a traffic ticket? My, my record’s horrible. They’ll send me to jail for something. I’m not doing it.

She revealed that she was not interested in these “liquid handcuffs.”

Personal experience with MAT was not always necessary for mothers to have reservations to this type of treatment. For example, Bella, a White mother in her 50s with one daughter, had no personal experience with methadone’s side effects and was a rare case of a woman in our study who had never used MAT. However, her husband had been using methadone, and she described how observing his experience deterred her from seeking methadone treatment:

I didn’t wanna do the methadone anyway only because I’ve watched people…I don’t like [husband] when he takes it. He gets really nasty, demanding, ordering, and if it’s not done his way you are degraded down to dirt, and I don’t like it anymore. I don’t like that methadone. When he doesn’t take it, he’s sick. So he doesn’t move around. He just stays in one frickin’ spot, curls up in a frickin’ ball and deals with it ‘til he gets down there to get it, because he did use to sell it.

Her husband’s reaction to using methadone served as a barrier to treatment for Bella who did not want to have similar experiences.

#### MAT Clinic Operating Hours

Medication-assisted treatment clinics often open early in the morning, sometimes at 5:00 am, and close mid-afternoon. This accommodates some patients who work in a traditional 9-5 job setting and have no transportation or housing issues but does little for those who work overnight and/or have transportation and housing challenges. Particularly mothers in caregiver roles struggle with the rigid schedules when they must juggle treatment and family obligations. In our sample of mothers, we found that the MAT clinics’ operating times can serve as a barrier to seeking treatment. Vicky, a White mother of three in her 50s, had previous treatment experiences with Narcotics Anonymous, detox centers, methadone and Suboxone clinics. Following her time in a methadone clinic, Vicky highlighted her reasons for not wanting to re-enroll:

Cause it’s a daily commitment; it’s a pain in the ass. You know what I mean? It’s every fuckin’ day you gotta go at 6:00 in the morning when I’d rather have a strip of medication that I can take when I want to, not because I have to. You know what I mean? Or be supervised to take it.

The hassle of going every morning to receive her methadone dose under supervision was too much of a hassle for Vicky who was in and out of homelessness during the past two years. Katie, who did not like the way methadone made her feel, concurred with Vicky in that the restrictive opening times presented a major hurdle to entering and continuing treatment. She shared:

Now in other states[…], one of my friends that lives up there. That clinic is open all day, you know, so you can go get your dose and you can take it before you go to bed. Now I may have had more success with it that way because it knocks you out. So, instead of having to take it in the morning and nod out at work all day, you know, you can go get it in the evening, fall asleep, take it so that by the time you wake up in the morning, you can actually get up and go to work.

Both women experience the opening times of the clinics as barriers to entering treatment.

Jessica was a White mother of two in her 30s who aspired to be completely sober, obtain a job, and purchase a car. As she worked toward her goal of quitting drugs, she was driven to the clinic by family members. Despite the familial support, however, Jessica described the struggles of the time and commitment to attending treatment:

Yes. And, you know, and I feel like that they should, you know - I don’t know if this had anything to do with it - give you more take homes. It is so hard to get up there every freakin’ day.

The clinic’s regulations do not allow for take-home bottles until patients have been with the program for a certain time and have been able to stay sober. For those that relapse, like Jessica, take-home bottles are out of reach, and she had to make the trip to the clinic every morning to receive her dose of methadone. The inflexibility presented a large barrier to mothers who were trying to stay sober.

### Treatment Facilities and Programs Barriers

Many of the barriers discussed by our respondents were tied to structural aspects of treatment facilities and programs, which were common to MAT as well as other types of treatment such as residential and outpatient programs. Women reported that it was difficult for them to find available treatment and they lamented the scarcity of programs designed to meet the special needs of mothers. When treatment was available the costs were often insurmountable without having access to Medicaid or private health insurance. To complicate matters for our participants who resided in the suburbs, treatment was often located in the cities and not easily accessible.

#### Access for Females

Treatment disparities for women are often exacerbated when women become pregnant and have children. Some women in our study indicated that their sex/gender acted as a barrier to entering treatment, and even more so when they became mothers. Lynette is a White mother in her 30s whose son who was removed by CPS. When we interviewed her, she was in search of employment to give her life “purpose” as well as to fulfill requirements requested by CPS necessary to regain custody of her son. She struggled to find a job and described that being a woman seeking treatment had limited her access to treatment compared to the resources and treatments available to men:

I don’t know, I’ve seen men get help better; like there’s more places for men. Women just have…it seems like they just have—like ‘cause they’re addicts they’re just washed up, used women. That’s what it seems like. Like there’s so many places for men, like I’ve seen it all around.

In the past, her family often paid for her treatment but now that Lynette was without that financial assistance, she struggled to find a place suitable for women that she could afford. She lamented that in her county “there’s one women’s spot and that it and the rest are men. And I’m like what the fuck?”

Katie echoed Lynette’s experience with accessing treatment centers focused on women, adding additional insight on barriers created by specific requirements:

I say it’s definitely more difficult because there are so many places that men can go to, especially homeless men. And there are some places that you know, if you’re a woman, you have to have a kid, but if you’re a single female with no children, good luck with your life.

While Katie found a program for women, it was only for women with children in their care, creating an additional barrier. For those mothers whose children had been removed by CPS, treatment access was made even more difficult, which in turn jeopardized the mother’s ability to abide by CPS imposed treatment regulations.

#### Cost

The costs associated with inpatient and outpatient opioid use treatment can stand in the way of seeking care. At times, our participants showed a willingness to seek treatment but could not get access to a program due to their financial situation. For example, the most accessible treatment is MAT, yet the costs of MAT vary from location to location as does the Medicaid coverage. As of 2018, through their respective Medicaid programs, all states reimbursed for some form of MAT but only 42 states paid for methadone treatment, for example (Substance Abuse and Mental Health Services Administration, 2018). At the time of data collection, Medicaid expansion was available in two states where we collected data, Connecticut and Massachusetts. Only one state, Georgia, did not have Medicaid expansion and had limited access to healthcare for those without private insurance. Not every participant in our sample qualified for Medicaid or had other access to health insurance, which exacerbated financial barriers to accessing MAT. Tess, a White mother of two in her 40s, recently stopped utilizing MAT after two years because of the mounting costs. Tess described her experience with comparing prices for MAT at different providers:

$11 per dose for the liquid and $12 for the tablet. And when we were in the trap house, we had called the one that was on [street]. The one with $11 and $12 was in [town], and the one on [street] said that they charged anywhere between $15 and $35 for a dose. And I said, so what if we’re homeless? And he was like, it’s $15 to $35 depending on your situation. And I was like, dude, I (laughs), I can get two days’ worth of heroin for what you’re charging for methadone. I’m going to find someplace. And then it was like $60 to start, and you didn’t get dosed that day. So I’m like, I can’t give you $60 and then have no money.

Tess situation exemplifies how the financial burden of paying for MAT can serve as a barrier to treatment. With a history of homelessness and unemployment and no access to health insurance, she was unable to afford entering methadone treatment and instead continued using the cheaper alternative, heroin.

Vanessa was actively trying to get on Suboxone as a form of MAT but she could not afford this treatment. For the past four years, she had been struggling with homelessness, incarceration, and lack of employment. She explained:

Suboxone’s retarded, and you have to get a prescription. First you got to find an actual doctor that will even mess with the shit, and then it’s like what, 4- or $500 each time you fill the fucker. Insurance doesn’t help, even if you had it. Suboxone is, unless you’ve got money, you’re not getting it.

This young woman serves as an example of someone who would be willing to try stopping her heroin use if only she could get access to a prescribing doctor and the medication. Her limited financial resources did not allow her to pay out-of-pocket for this treatment.

Treatment costs were not just associated with MAT but also with other types of treatments such as behavioral health treatment. Janet, a White woman in her 20s who was struggling with homelessness had utilized 12-step programs to help her stop injecting heroin. At the time of the interview, she had an appointment with an outpatient behavioral health center. This was not her first attempt at seeking treatment that went beyond a 12-step setting. She described her prior experience to find an inpatient behavioral treatment center: “I’ve tried to get into those facilities before and it’s—they told me either I have to have insurance or I have to have this amount of money.” Having neither, Janet continued going to her 12-step program, while desiring more targeted treatment to help her address the cause of her addiction.

#### Location

In our research, we found that opioid treatment and harm reduction resources were mostly located in the city, which impacted women living in the suburbs negatively. The ability to travel to treatment locations was often not part of the women’s realities when they also had to juggle childcare and job responsibilities. Valerie, a mother of two in her 50s, helped watch her grandson, which put her between one and two hours away from her MAT clinic. She noted that this set-up was “not convenient at all. […] which makes it hard.” Even when she did not stay with her daughter but in her own suburban apartment, the clinic was far away at the other end of the closest city, and it took Valerie several buses to get there.

Amanda, a White mother of two children in her 30s, was trying to regain custody of her children. One of the requirements imposed by CPS was that Amanda had to attend an Intensive Outpatient Program (IOP) several times a week which was not located close to her place of residence or work. Without access to a car and mounting bills for shared ride services, she struggled to attend regularly. She described the hardship the program’s location had created for her:

I have three meetings left to finish this IOP and that’s been a nightmare for me to get to [town]. I have to be there from 5:30 to 6:45 for this one meeting that I get nothing out of. I have to take the train and the train gets there at 3 so I have to kill two hours. I hate this. I literally have three to complete the service plan so I can at least say yeah, I did this.

There were no appropriate programs near where Amanda lived or worked. Treatment locations that require significant travel and time commitment posed barriers for women who were seeking help. In Amanda’s case, she was able to pay for the transportation that brought her to the IOP. Many women in our sample who struggled with housing and job insecurities would not have been able to comply with this CPS mandated treatment plan.

Whether or not treatment was available, women faced barriers due to the location of these treatment facilities. For example, one woman asked for a residential treatment facility to attend the day we interviewed her, but the only available bed was in a city area where she used to buy drugs. She was reluctant to go to this area for treatment. Our notes indicate that when we found a residential treatment bed for pregnant women or women with small children, and they did not want to go too far away, treatment professionals responded with stigmatizing allegations, such as “if she is not willing to go to another city, she doesn’t really want treatment.”

### Relationships Acting as Barriers or Motivators

Extensive barriers to treatment emerged from our interviews with suburban mothers. These included harmful relationships with romantic partners and family or friends. Being pregnant also emerged as a barrier to treatment in some cases, as did the relationship with CPS personnel. However, we also found that many of these same or similar relationships functioned as motivators to seek treatment. Primarily, relationships with people who are supportive were often critical for treatment success. The emotional, physical, and sometimes financial support provided through relationships can make a difference in the women’s decision to seek treatment and ability to participate in treatment.

#### Romantic Partners

Having a partner who uses or provides opioids can prevent women from seeking treatment. Often these partners make the drugs easily accessible, and sometimes they advise against treatment.

Tess, the White mother of two in her 40s who struggled with both the cost of MAT and staff attitudes at the clinic, rekindled her relationship with her current partner and described a promising beginning when he supplied her with methadone:

Well, first of all, when I went and met him after work, he gave me 10 milligrams, and I was literally just so excited to be around him that like the adrenaline. I probably could’ve quit everything and (laughs) been fine because I was just so on cloud nine.

Unfortunately, the emotional and happy reunion facilitated a transition back into heroin use, something Tess attributed to her husband: “If I had never got back together with [husband], I would’ve never touched heroin. [.] I probably either would’ve been on pills or weaned myself off, or done something about getting clean.” Her words demonstrate that Tess’s relationship with her partner was a barrier to her seeking treatment.

Rebecca’s experience supported Tess’s story. When Rebecca could not afford drugs, she would ask her husband and father of her children to provide drugs for her, and he obliged. These romantic relationships effectively stood in the way of seeking treatment.

Romantic relationships were not always obstacles to entering treatment. Some romantic relationships surfaced as a mode of encouragement for mothers to seek treatment for their opioid dependence. Women who had partners to support them emotionally and who were supportive of their treatment were found to seek treatment more often than women who had partners that either used opioids, were abusive, or both.

Jennifer was a Latina with two children in her 40s who was able to stop using prior to getting pregnant with her twins. She explained: “Yes, we got clean together, and he’s still clean right now.” Her partner supported her decision to stop using drugs by joining the effort. Jennifer was able to stay sober for over 10 years when she was raising her sons and only relapsed when she lost her children to her partner.

Like Jennifer, Janet also had a partner who supported her cessation efforts. Janet was a White mother in her 20s who previously had two years of sobriety, describing this time as the “happiest I ever was in my entire life.” However, Janet relapsed when she lost her job and car. Despite these obstacles, she remained resilient, and she and her boyfriend sought detoxification together: “But me and [boyfriend] are very serious so we’re trying. We both—we didn’t wanna leave each other, but we knew we were gonna be separated for detox.” Both prioritized treatment over being together and supported each other through the first step of this process with the detoxification program.

#### Family and Friends

Similarly, relationships with family and friends can also act as a barrier or a motivator to seeking treatment. Typically, women’s narratives revealed more hindrances to treatment due to family and friend relationships. Hardships with the family or difficulties with friends often made treatment not a priority. Attitudes of friends who use opioids, such as “I like you better when you’re high” stopped some mothers from seeking help for fear of losing their social circle. Losing a supportive social network and feeling isolated and lonely kept respondents from engaging in treatment.

Abby, the White mother of four in her 30s who was motivated to move to the suburbs to access a mobile Suboxone unit, was currently homeless and temporarily sleeping in a park gazebo with the winter looming. Abby lost her mother and stepmother to cancer and primarily had using-friends, who she referred to as “backstabbers.” Abby summarized how she felt about her current situation: “Cause there’s nothing for you to do, and you’re trying to stay clean but it’s really hard; trying to stay clean and to do the right thing when you don’t have the right support systems.” With no positive relationship in her life, Abby found the obstacles of entering treatment insurmountable.

Vanessa, the mother described previously, also suffered from having difficult relationships. Vanessa’s father walked out on her family at an early age, and her mother was emotionally abusive. Her few non-using friends lived in another state and were unaware of her relapse into heroin use. During a year of treatment seeking, Vanessa described strains in her relationships: “Me and her [Vanessa’s wife] were busting our ass and all of our friends turned their back on us, and nobody wanted to help us out.” Aside from her wife, Vanessa did not feel supported: “Like I’m so fucked right now, and nobody will help me and nobody cares, so why should I care about myself if nobody else gives a damn? What the hell do I have to fight for, then?” Vanessa’s situation highlights the predicament of having difficult relationships with family and friends. The feeling of being alone and not cared for in this life present barriers to wanting to make changes such as entering a treatment program.

Lynette echoed Vanessa’s feelings of feeling unsupported. She recently moved from another state and found herself without a supportive network of 12-step friends that she had for more than 10 years. She described how helpful they have been in the past:

[I]f I ever need anything or need treatment or anything, they’d help me. Like up here it kinda—‘cause I’m so far away from anyone I have like that it kinda—I’m not held accountable. So when I use up here I, you know, I…kinda—I use a lot differently than I would in Florida when I’m around them.

Being isolated from positive social interactions, Lynette started using heroin again.

Relationships with family and friends are not categorically barriers to treatment. We found that similar to having a supportive romantic partner who motivates treatment seeking behaviors, some women have non-using family members and friends who encourage them to pursue treatment. Rita, a Black woman in her 60s, recently moved from another state with her husband. She was staying with her daughter in the suburbs and relapsed with her husband. She recounted what motivated her to enter treatment:

We were clean when we came down here. So then we found [city] and that’s where we started sneaking to get drugs, you know, because we was living with my daughter, so we couldn’t just out, we’d get high, you know. So we were sneaking. So then she sat me down one day and she said, I will take your ass to the bus stop, put you on the bus and get you out of here. “You either decide to stay clean now, or get out my house.” And so I said, “well that don’t sound too good, so I decided to get clean.”

Her daughter had been a positive influence in Rita’s life for years, allowing her mother to stay when she did not have a home or helping her find treatment throughout the years. The strict rules she imposed motivated Rita to seek treatment in order to keep her housing and be able to see her grandchildren.

Not every non-using family member offered as much assistance as Rita’s daughter. Others took a different approach to motivate their loved ones to enter treatment. Before Valerie entered MAT treatment, she was homeless. Turning to her daughter, she described what happened next:

She turned me—‘cause usually my family don’t turn me down. And I went to my daughter’s house and she turned me down. Told me, “Mom, you can’t stay here.” That really, you know, put a burden on me. I mean like somebody took a knife and just stabbed me in my heart ‘cause she—depend—don’t matter how I looked at—she always opened the door for me.

Valerie considered this her “breaking point.” Her daughter, who was always there for her, denied her help. As Valerie put it: “They got tired of it.” This incidence motivated Valerie to seek treatment.

Annie, who lost two children in tragic ways, recounted how her 12-year-old daughter motivated her to go to treatment for seven months:

And then my daughter, her birthday was in [month], and I was askin’ her what did she want for her birthday and she said she want—she was like, ‘Mom, I just want you to live.’ So, I did, and I went to treatment.

At the same time, Annie also was supported by a nurse who befriended her when she spent time at the hospital before her son passed away.

Moving away from drug using friends and acquaintances was a reoccurring theme in our sample. These friendships were unsupportive of treatment and encouraged further drug use. Katie, a White woman in her 30s who just suffered her fourth miscarriage, moved out of her hometown because many in her social circle were using drugs. She describes her move to the suburbs where she has helpful friend relationships:

That was a quick fix. Out here, you know, it’s like I have enough friends out here. And it’s just comfortable. It’s quiet, you know? Um, I’m close to anything that I need but I’m far enough away…from any bad shit that it would be like a real pain in the ass if I decided, oh I wanna go get high.

Being removed from relationships that would encourage drug use, and finding new friend in a new place, helped Katie to abstain, which shows the positive influence of supportive relationships on drug using behaviors.

Other women described ways that non-using friends motivated them to enter treatment for their opioid use. For example, these friends introduced non-drug related activities that were attractive to those seeking recovery. Amanda, who was trying to regain custody of her children, shared her thoughts on the social benefits of 12-step meetings:

AA and NA allow you to get back into the real world, meet sober people, start doing kind of more normal things. Let’s go get coffee, let’s go to the movies. They have different things going, like a sober dance. You have to find the meetings you like, like some people don’t like the war stories they don’t want to hear about how great and how crazy or whatever. I try to look for uh—like more the ones that talk about recovery, what they did to keep themselves clean the last twenty years.

She recounted that she spent years in isolation because all she could concentrate on was how she would get money to buy drugs. This took up the vast majority of her time. Gaining access to a group of non-using friends who engaged in fun activities was a motivator for Amanda to seek out and continue with this type of treatment.

#### Pregnancy

Pregnancy was revealed to also be both a motivator and barrier to seeking and enrolling in treatment. The relationship our participants had with the unborn child influenced treatment decisions because of potential treatment side effects. However, women’s access to treatment was affected by their pregnancies. For example, Mallory did not believe it was best for her unborn child to be exposed to Suboxone while pregnant, “because I just think it’s awful. It’s not worth it. […] I don’t think it’s good for a baby to get…be born that’s kickin’ Suboxone like that […] ‘Cause if I can’t, I don’t think a baby can.” To complicate matters, when Mallory tried getting into detox she described being denied entry because of her current pregnancy: “I tried and they told me that—that’s when I found out that I was pregnant. […] They kicked me out. […] They said they don’t deal with pregnant women.” When the interviewer offered helping Mallory find a place to detox, Mallory said “Oh I’d go in a second.” The inability to get into detox due to her pregnancy and her current use made Mallory consider terminating her pregnancy, “Like I don’t even know if I’m gonna keep this baby.” Mallory’s pregnancy was inadvertently a barrier to entering treatment.

While we found that pregnancy could prevent treatment, we also discovered that pregnancy functioned as a motivator for entering treatment. Many of our participants indicated that as soon as they discovered their pregnancies, they either discussed treatment options with their providers or they detoxed with the help of professionals. For example, Carol, a White woman in her 50s and mother of a daughter who resided with her father in a different state, explained:

When I found out I was pregnant I went immediately into detox and got detoxed and then I just stopped ‘cause I did not wanna have my child be born on any kind of meth—I’ve seen methadone babies and I’ve heard about it and I didn’t want anything to be wrong with my daughter. And…when my daughter was born, just the love I felt for her was—I—you know how it feels. You know how it feels.

The above quote exemplifies the mindset that many women who discovered they were pregnant while using opioids had. Believing that methadone could harm the unborn was a common theme and sometimes resulted in women withdrawing from opioids without MAT. Carol made it very clear that she had the well-being of the child in mind and that her pregnancy motivated her to detox immediately.

Contrary to Carol’s fear of methadone and its unintended consequences for her unborn, Amanda, a White woman in her 30s took her doctor’s recommendation to heart. The mother of two, who had lost custody of her older son to the father, recently gave birth. When she got pregnant for the second time, she decided to seek treatment after conversations with her medical provider. She explained:

But when you get pregnant they scare the crap out of you, they say you cannot stop using, you have to continue this program, because I was a heroin addict at that point. They said I had to continue doing something whether it be Suboxone or methadone, you can’t stop using. […] if I just stopped cold turkey, I could miscarry. So they pushed me over to the subutex. Of course they have no blocker so I was kind of abusing here and there. So they said I needed a higher form, so they put me on the methadone. Which is good, I stopped using, I wasn’t using.

Suboxone did not work for Amanda and she continued using opioids. Methadone, however, allowed her to come off the opioids successfully. At the time of the interview, she was working with CPS to gain custody of the newborn.

#### Child Protective Services

Child Protective Services (CPS) aim to work with families and communities to keep children safe from abuse and neglect. In many cases CPS is able to provide support and services to keep children safe with parents or family members. CPS provides foster care or finds new permanent families for children through kinship, guardianship, or adoption if the need arises. However, for many mothers who use opioids, there is a constant worry that CPS administrators or staff will judge them unfit to parent their children effectively. While CPS involvement can motivate some mothers to enter treatment in order to keep or regain custody of their children, participants in our sample were clear that it could also have the opposite effect on them. The fear of involving the social service agency drove some to hide their drug use from everyone and avoid any type of treatment.

Tiffany, who earlier expressed having negative perceptions of MAT use, also feared involvement of CPS. The agency had never been involved in her life, and she considered herself lucky that “their father always picked up. If I fell, he picked up.” Tiffany knew what it felt like to have access to her children denied when her husband took her daughter away from her the last time he found out she was using drugs. She ended up being homeless until she became pregnant again. This fear of losing her children drove her to hide her drug use even when she saw her primary care physician. She explained her reasons for keeping her use a secret: “No, ‘cause I don’t want nobody callin’ [CPS] on me or anything on me, and I feel like that’s what’ll happen.” Her fear resulted in Tiffany not entering any type of treatment. She felt that if CPS became involved in her life she “would probably lose it.” In her situation, potential CPS involvement acted as a barrier to treatment.

The threat of having a child removed by CPS loomed large in the lives of mothers who use opioids. Nevertheless, while anxiety can lead to drug use and mental health problems, we found that in some cases, the anxiety associated with CPS intervening in a family can be great enough to motivate some women to seek treatment.

Annie was a White woman in her 30s and a mother of four. She saw herself as a “functioning addict” and in her own words “tries to keep up appearances” when she is out and about in the neighborhood. She describes her interaction with CPS:

They done urine screens, they done hair follicle tests. Because my urine screens were good and I told them, like, “this is what I’m doin’. I’ll go to treatment. I don’t mind. I’ll do treatment, but you’re not taking my kids. Tell me what to do, and I’ll do it.” So that’s how—my approach to it was always, “Alright, tell me what to do and I’ll do it.”

Annie’s quote shows that some women were very willing to enter treatment in order to keep their children. Because she met the terms of all CPS mandates, Annie never lost custody of her children. She always complied and entered treatment instead. She stayed sober throughout her last pregnancy and at the time of the interview had an eight-week-old daughter in her custody. She just recently relapsed on the five-year anniversary of one of her son’s death.

Our field notes indicate that some women went so far as to attempt having their children at home rather than give birth at a hospital for fear of losing the baby to CPS. These women were risking childbirth complications in order to avoid contact with and punishment by social services.

## Discussion

This is the first qualitative study investigating barriers and facilitators to treatment among mothers and pregnant women who use opioids living in suburban environments. Our findings build on previous literature showing that both stigma and lack of access due to structural factors are significant barriers to treatment (Redko et al., 2006; Wisdom et al., 2011; Hammarlund et al., 2018; Kahn et al., 2019; Abraham et al., 2020; Acevedo et al., 2020; Nichols et al., 2021), which are exacerbated for women who are pregnant or mothers (Howard, 2015; Stone, 2015; Angelotta et al., 2016; Whittaker et al., 2016, 2019; Lee and Boeri, 2017; Frazer et al., 2019; Lamonica et al., 2021).

The barriers caused by the stigma of MAT, including attitudes from service professional staff and community, were reinforced by perceptions the women had about MAT from their own experiences or experiences they heard from others. While the social stigma associated with MAT is changing as public education on the success of MAT to combat rising overdose death rates increases (Heavey et al., 2018; Irvine et al., 2018; Silverstein et al., 2019; Adams and Volkow, 2020), institutionalized and public stigma of mothers or pregnant women who use opioids is still prevalent (Stone, 2015; Nichols et al., 2021). Being seen entering a MAT clinic increases the chances that such women will be discredited by the community (Goffman, 1959; Chaudoir et al., 2013), and disapproving attitudes of some providers toward pregnant women who use opioids remain.

Previous research shows increased stigma in rural areas toward people who use opioids, resulting in less support for harm reduction initiatives in rural and non-urban areas (Borders and Booth, 2007; Childs et al., 2021). Similarly, the suburbs are often viewed as having fewer drug use problems than urban areas, thereby increasing stigma of drug use and decreasing the availability of treatment. Barriers related to accessing treatment facilities included distance to the locations, compounded by lack of public transportation, costs for treatment, hours of operation, and few treatment programs for women with children. Location, waiting lists, and cost of treatment were common barriers to seeking MAT, outpatient, or residential treatment. Lack of places where mothers could live with their children were barriers for women seeking residential programs. These findings add to extant literature showing geographical obstacles and a dearth of treatment for women are barriers to treatment seeking (Marsh et al., 2000; Paltrow and Flavin, 2013).

Research on rural areas found that fewer treatment options, social stigma, and lack of transportation create barriers to treatment adherence (Amiri et al., 2018; Childs et al., 2021). Research in urban areas found that in addition to stigma, fear of losing custody of children and loss of relationships with partners were barriers specific to pregnant women (Whittaker et al., 2016; Frazer et al., 2019). Our findings confirm that the barriers common in rural and urban areas are also barriers for women living in the suburbs. However, these barriers differed by structural aspects, such as lack of access due to costs of treatment and transportation. For example, women in suburban Atlanta, Georgia, where MAT was virtually non-existent at the time and there is no public transportation to the city, had very limited access to treatment.

Social stigma was experienced by women in suburban areas in all three states, including those where health insurance and services were widely available. Like women in rural and urban areas, fear of losing custody of children and separation from family and partners were critical barriers to treatment-seeking expressed by all women in all three suburban areas regardless of the state. Consistent with studies on treatment-seeking in urban and rural areas, our suburban study revealed the impact of social stigmatization on pregnant women and mothers who use opioids was a common barrier, while other barriers were structural (Pollini et al., 2006; Redko et al., 2006; Wisdom et al., 2011; Harris and McElrath, 2012; Hammarlund et al., 2018; Kahn et al., 2019; Acevedo et al., 2020).

Informed by a triangulation of stigma and life course theoretical frameworks (Goffman, 1959; Elder, 1999; Laub and Sampson, 2003; Chaudoir et al., 2013; Howard, 2015), we suggest that many of these barriers can be addressed by targeted structural changes. These include policy modifications that focus on reducing institutionalized stigma by decreasing blatant surveillance and providing more compassionate care for women of child-bearing age who are opioid dependent. This is most evident in how service providers convey messages that stigmatize women’s relationships. Women who are pregnant or caring for small children are often emotionally and financially dependent on their relationships with others. Without acknowledging the women’s intimate relations with family and partners, including financial reliance, efforts addressing opioid dependence among pregnant women and mothers will unintentionally construct barriers to seeking treatment. Our data show that when women are asked to abandon relationships or suggested to terminate partnerships, they are often overwhelmed with emotional stress or economic burdens that hinder treatment-seeking.

We add to the literature not only by providing insights on suburban women who use opioids, but also by disentangling barriers that can be addressed structurally from those that are entwined as potential barriers and/or motivators, specifically relational factors impacting treatment-seeking behavior. We know stigma related to MAT and perspectives of MAT are changing in the public view due to the opioid crisis (Adams and Volkow, 2020). Moreover, the structural barriers we identified regarding facilities can be addressed through policy change, such as increased funding for residential treatment exclusive to the needs of pregnant women and mothers, and consideration of location and operating hours of treatment facilities. However, the relational barriers discussed here need more research to be fully addressed. Women’s relations with romantic partners, family or friends can motivate them to seek treatment or they can be a barrier, which often is contingent on the social context of the relationship, as well as the mental, emotional, or economic situation of the women. While relational factors have been examined in previous studies, research often focuses on relations that act as barriers to treatment (Marsh et al., 2000) or relations that act as motivators (VanDeMark, 2007). Rarely is analysis focused on both relational barriers and motivational influences (Frazer et al., 2019). Our findings suggest that relations that act as barriers can be transformed to potential motivators for the women if intervening factors such as emotional and mental health are assessed, family situations are acknowledged, and financial resources are provided.

Our findings support studies showing that more effort is needed to reduce real and perceived stigmatization of pregnant women and mothers who use opioids (Nichols et al., 2021). Empathy, compassion, respect, and support provide greater treatment-seeking motivation among opioid-dependent women with children than the current focus on supervision and surveillance (Howard, 2015; Stone, 2015; Adams and Volkow, 2020). We enhance the findings of these studies with evidence provided by our life course examination of the women’s experiences over time. Their lives show that punitive and moral-focused policies have resulted in barriers to treatment as well as potentially creating obstacles to intact families where mothers can remain together with their children and partners. While institutional and structural changes are needed to address economic and geographic logistical difficulties to treatment, providers working directly with women through social and healthcare services can go a long way in helping reduce social stigma and fears of losing children and intimate relationships. Our analysis provided insights on life course patterns of relationships that suggest social and emotional processes must be considered when designing programs for opioid dependent pregnant women and mothers with children in their care (Giordano et al., 2007). Consistent with findings on the social bonding aspects of life course theory (Laub and Sampson, 2003), women’s emotional relationships can be a barrier or a motivator to seeking treatment for opioid use. The current focus on surveillance may be counter-productive if the relationships that pregnant women and mothers have with children, family, friends, and partners are not taken into consideration.

### Limitations

This study was limited by a relatively small sample compared to quantitative studies; however, a sample of 58 participants is large for qualitative studies. Qualitative findings are not meant to be generalizable but to provide in-depth and detailed information that can inform large scale studies to test the results. While we achieved diversity in terms of drawing from a range of geographic locations, the small sample size in each location does not adequately represent diverse racial and ethnic populations, and an over-sampling of pregnant women and mothers who are African American/Black, Latina, and other ethnicities is desirable. Finally, our study is limited by including the perspectives of only one side of the relationships between public agencies and pregnant women or mothers who use opioids. Studies including all actors in this relationship are needed, as well as studies of custodial fathers who use opioids.

While we used a life course analysis to examine current as well as historical barriers and motivators to treatment-seeking behavior among pregnant women and mothers who use opioids, we acknowledge that continuing Medicaid expansion provided by the Affordable Care Act will help to address some of the barriers found here. However, health insurance is not a panacea for the widespread stigmatization of opioid-dependent pregnant women and mothers, and the nearly hegemonic call for increased surveillance. Our study shows the need for less surveillance and a greater focus on emotional aspects of mothering can provide motivation rather than barriers to treatment-seeking.

### Future Research

Our findings suggest more research is needed on ways to increase treatment-seeking motivators for mothers and pregnant women. Treatment research suggests that treatment motivation is a predictor for remaining in treatment (Rapp et al., 2007; Hiller et al., 2009). Many women thought they were not motivated for treatment, although our in-depth inspection of their narratives uncovered personal problems connected to seemingly unsurmountable hardships attributed to their lack of motivation (Pollini et al., 2006; Acevedo et al., 2020). While previous studies show there is critical time for treatment motivation, the responsibility is often on law enforcement, social services professionals, and treatment providers to assess the need for treatment, as well as identify barriers that hinder access to treatment (Binswanger et al., 2011; Kahn et al., 2019). Good intentions, such as increased surveillance by these agencies, can result in unintentional barriers to seeking necessary treatment (McMahon et al., 2002; Paltrow and Flavin, 2013; Olsen, 2015; Angelotta et al., 2016; Frazer et al., 2019; Honein et al., 2019). More research is needed on how health and social services providers, who are the first contact with mothers, might practice motivational interviewing skills with mothers and newly pregnant women (Mullins et al., 2004). Studies are needed to identify links between emotional and social processes, how these processes are impacted by structural disadvantage (Giordano et al., 2007), and how emotional relationships can be used to initiate new lines of action (Collins, 2004) among opioid dependent women.

While our finding on the interactional effect of stigma, structure, and emotional relationships was an emerging result of a triangulation analysis, how to address this is beyond the scope of our paper and left for further research. Research also is needed on how peer support services and shared decision making might increase motivation by identifying and addressing emotional and relational barriers (White, 2004; Rigg and Murphy, 2013; Kahn et al., 2017). More studies are needed on peer support throughout the course of opioid treatment and beyond, how peers might identify structural disadvantages that intersect with social relations and reveal the emotional dynamics that serve as motivators or barriers to treatment (Giordano et al., 2007). Research at the institutional level is needed to examine the effect of more supportive care practices versus surveillance as social control mechanisms. Studies at the structural level are needed to identify how to implement more humane and compassionate policies in contrast to moral policies governing pregnant women and mothers who use opioids (Whittaker et al., 2019).

## Data Availability Statement

The raw data supporting the conclusions of this article will be made available by the authors, without undue reservation.

## Ethics Statement

The studies involving human participants were reviewed and approved by Southern Connecticut State University IRB. The patients/participants provided their written informed consent to participate in this study.

## Author Contributions

AP, GM, and CB conducted the preliminary analysis and wrote a first draft of this manuscript with MB, who helped guide the conceptualization. AL and JT contributed to the analysis with additional data and enhanced conceptualization. AK wrote the Results. MB contributed to the organization of the results and enhanced conceptualization, wrote the Introduction, Methods, and Discussion. MB and AK worked together on revisions and editing and approval of the final manuscript for submission. All authors contributed to the article and approved the submitted version.

## Conflict of Interest

The authors declare that the research was conducted in the absence of any commercial or financial relationships that could be construed as a potential conflict of interest.
